# Minimally invasive follicular thyroid carcinomas: prognostic factors

**DOI:** 10.1007/s12020-016-0876-y

**Published:** 2016-02-08

**Authors:** Gustav Stenson, Inga-Lena Nilsson, Ninni Mu, Catharina Larsson, Catharina Ihre Lundgren, C. Christofer Juhlin, Anders Höög, Jan Zedenius

**Affiliations:** Department of Molecular Medicine and Surgery, Karolinska Institutet, 171 77 Stockholm, Sweden; Department of Breast and Endocrine Surgery, Section of Endocrine and Sarcoma Surgery, Karolinska University Hospital, 171 76 Stockholm, Sweden; Department of Oncology-Pathology, Karolinska Institutet, CCK, Karolinska University Hospital, 171 76 Stockholm, Sweden; Sophiahemmet Hospital, 114 86 Stockholm, Sweden

**Keywords:** Follicular, Thyroid, Carcinoma, Minimally invasive, Prognosis

## Abstract

Although minimally invasive follicular thyroid carcinoma (MI-FTC) is regarded as an indolent tumour, treatment strategies remain controversial. Our aim was to investigate the outcome for patients with MI-FTC and to identify prognostic parameters to facilitate adequate treatment and follow-up. This retrospective follow-up study involved all cases of MI-FTC operated at the Karolinska University Hospital between 1986 and 2009. Outcome was analysed using death from MI-FTC as endpoint. Fifty-eight patients (41 women and 17 men) with MI-FTC were identified. The median follow-up time was 140 (range 21–308) months. Vascular invasion was observed in 36 cases and was associated with larger tumour size [median 40 (20–76) compared with 24 (10–80) mm for patients with capsular invasion only (*P* = 0.001)] and older patients [54 (20–92) vs. 44 (11–77) years; *P* = 0.019]. Patients with vascular invasion were more often treated with thyroidectomy (21/36 compared to 7/22 with capsular invasion only; *P* = 0.045). Five patients died from metastatic disease of FTC after a median follow-up of 114 (range 41–193) months; all were older than 50 years (51–72) at the time of the initial surgery; vascular invasion was present in all tumours and all but one were treated with thyroidectomy. Univariate analysis identified combined capsular and vascular invasion (*P* = 0.034), age at surgery ≥50 years (*P* = 0.023) and male gender (*P* = 0.005) as related to risk of death from MI-FTC. MI-FTC should not be considered a purely indolent disease. Age at diagnosis and the existence of combined capsular and vascular invasion were identified as important prognostic factors.

## Introduction

Follicular thyroid carcinoma (FTC) today accounts for about 10 % of all thyroid cancers in iodine-sufficient populations [1]. Together with papillary thyroid carcinoma (PTC), by far the most common thyroid malignancy, FTC constitutes the so-called differentiated thyroid cancers of follicular origin. It is more common in women and its highest incidence is reported to be shortly after menopause [2–4]. FTC is generally subdivided into widely and minimally invasive FTC (WI-FTC and MI-FTC) [5]. MI-FTC is limited to microscopic capsular and/or vascular invasion, described in the WHO classification [5] as “focal, minimal, a focus, limited, a single focus”, whereas widely invasive is described as “extensive, widespread invasion, multifocal areas, multiple foci and extensively invaded”. FTC is generally considered to have a good prognosis, especially in young patients [6]. This is particularly true for MI-FTC [2, 6]. Despite this, some cases metastasise and/or develop local recurrence [2, 4].

As the diagnostic criteria are based on histopathology [5], FTC cannot be differentiated from follicular thyroid adenoma by fine needle aspiration. Some molecular alterations could aid in differentiating between FTC and PTC. The *PAX8/PPARγ* rearrangements is found exclusively in follicular neoplasms, while the *RET/PTC* rearrangements are exclusive for PTC [7–9]. *PAX8/PPARγ* rearrangements, RAS mutations and *TERT* promoter mutations are recurrently seen in both MI-FTC and WI-FTC [10, 11]. In addition some genetic alterations, e.g. loss of heterozygosity for *HGF,* have been observed in MI-FTC only [12]. The treatment of MI-FTC is still debated; many advocate lobectomy only, mainly to reduce surgical complications [3, 6]. A further subdivision of MI-FTC into cases with capsular invasion only and those with vascular invasion has been proposed, but previous studies have been contradictory [3, 6, 13]. Although novel data may aid in the preoperative decision-making, we today have no sufficient tools to discriminate those patients that would benefit from more extensive treatment, although new molecular panels for fine needle aspirates have been proposed [14]. Still, several clinical features should be taken into account for the individual patient [15].

The aim of this study was to characterise the prognosis of patients with an initial diagnosis of MI-FTC and to identify prognostic parameters to facilitate clinical decision-making for adequate treatment and follow-up.

## Materials and methods

Databases from the Department of Surgery and Department of Pathology together with histopathological reports were used to identify all cases of minimally invasive FTC (MI-FTC) operated between 1986 and 2009 at the Karolinska University Hospital, Sweden. The tumours were re-classified according to the most recent WHO criteria [5] by one pathologist. Only FTCs with limited capsular invasion and/or limited vascular invasion were selected. Patients with atypical follicular adenomas, WI-FTC, follicular variant of PTCs, or poorly differentiated (insular) tumours were excluded. Metastasis at the time of diagnosis was not an exclusion criterion, provided the FTC was classified as MI-FTC on the basis of the thyroid specimen per se.

Fifty-nine patients with MI-FTC were identified. One was lost to follow-up due to emigration shortly after surgery. All clinical data and histopathology reports of the remaining 58 cases were re-evaluated and collected for analysis. Patient and treatment data, tumour size, invasion pattern and tumour type were recorded. Presence of >75 % oncocytic cells was the prerequisite for diagnosis of oncocytic tumours (i.e. Hürthle cell carcinoma). Proliferation data (determined by immunohistochemical analysis of Ki-67) was available for cases operated after 2002 only, and was therefore not included in the analysis. The type of surgery was registered as total thyroidectomy or lobectomy, where supplementary lobectomy within a month after the original operation was classified as total thyroidectomy. If tumour cells were found anywhere at the tumour resection margin upon histopathological examination, the resection was judged to be microscopically non-radical. Thyrotropin (TSH) suppressive treatment with thyroxin, adjuvant treatment with radioactive iodine, presence of lymphatic spread and distant metastatic disease at diagnosis, as well as recurrent disease, both local and metastatic, were recorded. The decision on the extent of the surgical procedure and adjuvant treatment did not follow a distinct protocol; it followed the judgement of the responsible clinician during the study period. Follow-up time was calculated from the time of the primary operation until September 2015 with the exception of one patient, who was followed until emigration in 2002.

The study was approved by the local ethics committee. All patients gave their informed consent for collection and analysis of tissue material and clinical data at the time of treatment.

## Statistical analysis

Statistical analysis was performed with the IBM SPSS Statistics 23.0. 0.0 (Armonk, NY, USA). Data are expressed as median and range. The primary endpoint was death from FTC. Analysis of survival and prognostic significance of clinical parameters were performed with the Kaplan–Meier method and the log-rank test. *P* values ≤0.05 were considered statistically significant. The Cox regression model was used for multivariate analysis to identify independent prognostic factors [16].

## Results

Clinical, histopathological and follow-up data for the 58 patients with MI-FTC are summarised in Table 1. *P* values obtained from the statistical analysis of death rates are included. Of the 58 patients, 41 were women (71 %) and 17 men (29 %), with a median age at diagnosis of 50 (range 11–92) years and a median tumour size of 35 (range 10–80) mm. Vascular invasion was observed in 36 cases (62 %) and was associated with larger tumour size [median 40 (range 20–76) compared with 24 (10–80) mm for patients with capsular invasion only; *P* = 0.001] and older patients [54 (20–92) vs. 44 (11–77) years.; *P* = 0.019]. Isolated capsular invasion was observed in 22 cases (38 %) and isolated vascular invasion in 5 cases (9 %).

**Table 1:** Patients with minimally invasive follicular thyroid cancer, clinical characteristics and analysis of the significance of mortality related to thyroid cancer

Parameter (no. of informative)	Cases observed		Death rate^a^		*P* value
	No.	(%)	No.	(%)	Log-rank test
Age at surgery (*n* = 58)					
≥50 years	29	50	5	17	0.023
<50 years	29	50	0	0	
Gender (*n* = 58)					
Female	41	71	1	2	0.005
Male	17	29	4	24	
Tumour size (*n* = 58)					
<20 mm	4	7	0	0	0.820
20–40 mm	37	64	4	11	
>40 mm	17	29	1	6	
Hürthle (*n* = 58)					
Yes	18	31	0	0	0.133
No	40	69	5	22	
Capsular and vascular invasion (*n* = 58)					
Yes	30	52	5	17	0.034
No	28	48	0	0	
Capsular invasion (*n* = 58)					
Yes	53	91	5	9	0.457
No	5	9	0	0	
Vascular invasion (*n* = 58)					
Yes	36	62	5	14	0.085
No	22	38	0	0	
Surgical margin (microscopical) (*n* = 58)					
Radical	56	97	4	7	0.080
Non-radical	2	3	1	50	
Operation type (*n* = 58)					
Lobectomy	30	52	1	3	0.176
Total thyroidectomy	28	48	4	14	
Ablative treatment (*n* = 57)					
Given	18	32	4	22	0.023
Not given	39	68	1	3	
TSH suppressive treatment (*n* = 57)					
Given	45	79	3	7	0.781
Not given	12	21	2	17	
Recurrence (*n* = 58)					
Total	5	9	5	100	
Metastases	5	9	5	100	
Loco-regional	1	2	1	100	
Mortality (*n* = 58)					
Dead from disease	5	9			
Dead from other causes	8	14			

^a^Death rate refers to patients who died from FTC

Twenty-eight patients (48 %) underwent total thyroidectomy; the rest underwent lobectomy. Total thyroidectomy was more common among patients with vascular invasion (21/36 compared to 7/22 with capsular invasion only; *P* = 0.045). Eighteen of 57 patients (32 %) with available data on adjuvant treatment had received ablation of the thyroid remnant together with radioactive iodine therapy; 45/57 patients (79 %) had received TSH suppressive treatment at least 5 years post-operatively.

**Fig. 1:**
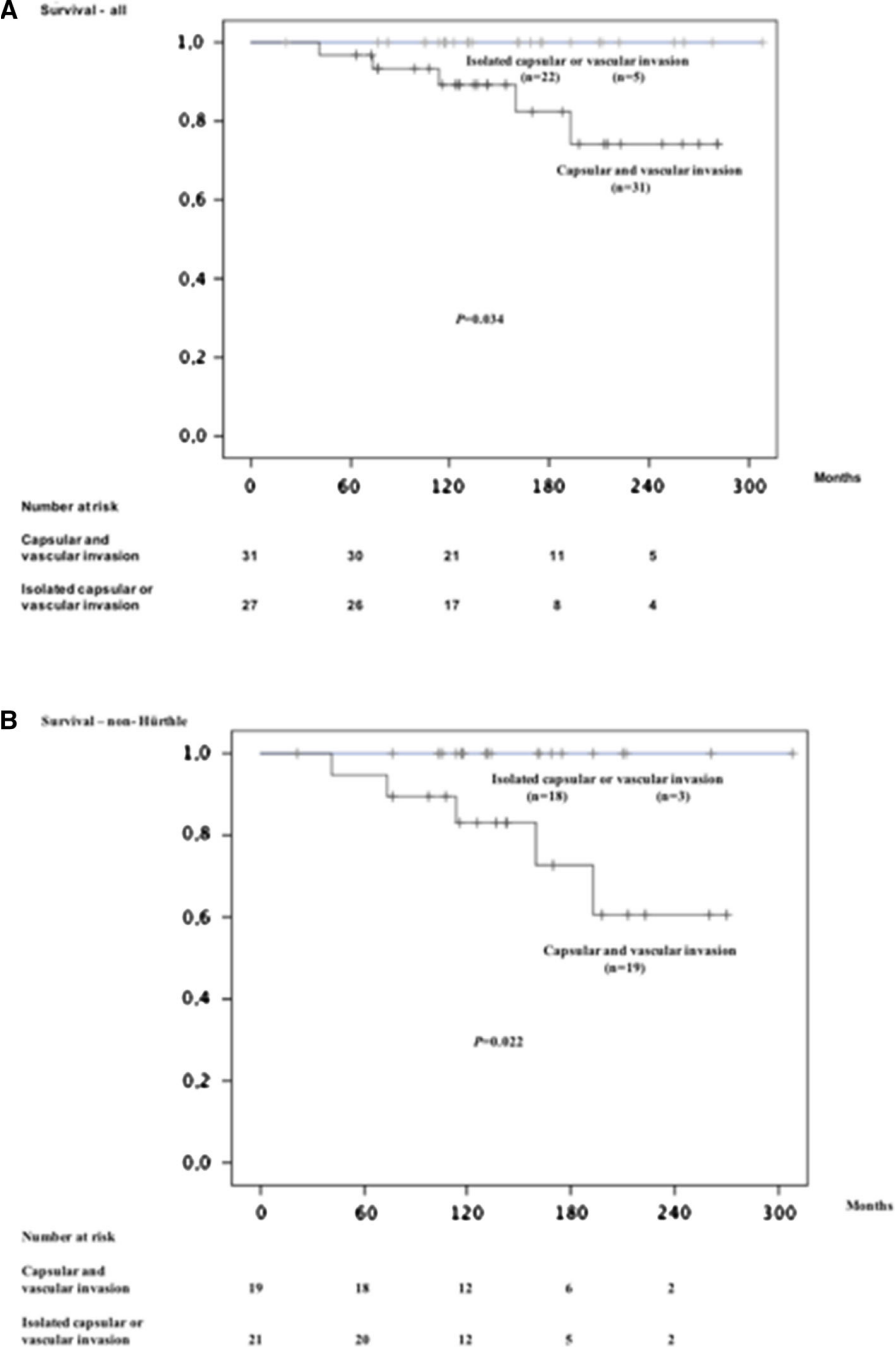
Kaplan–Meier plot illustrating disease-related survival related to combined vascular and capsular invasion among the 58 MI-FTC patients (**a**) and among the subgroup with non-Hürthle cell carcinoma (**b**). *Short vertical lines* indicate censored individuals. *P* values were calculated using the log-rank test. Below each panel is detailed the number of patients at diagnosis (0 m) and at different time-points during follow-up (60, 120, 180 and 240 months)

The median follow-up time was 140 (21–308) months. Eight patients died from causes other than FTC and had no signs of recurrence, with a median follow-up of 140 (21–176) months. Five patients died from FTC after a median follow-up of 114 (41–193) months. The median age at diagnosis was 57 years (51–72) for these patients; median tumour size was 35 (30–50) mm. The patients who died from FTC all presented with tumours exhibiting both vascular and capsular invasion (Fig. 1a). All developed recurrent disease; two of them were diagnosed with distant metastasis already at the time of diagnosis. No patient had signs of lymphatic spread. Four of these five patients had undergone total thyroidectomy and one of them was classified as microscopically non-radical (tumour cells found in the resection margin).

Univariate analysis identified male gender (*P* = 0.005), age at surgery ≥50 years (*P* = 0.023) and co-existing capsular and vascular invasion (*P* = 0.034) as related to postoperative metastatic disease and death from this disease. Gender was the only independent prognostic covariate when applying multivariate analysis (*P* = 0.035). No patient with tumours predominantly consisting of oncocytic cells (Hürthle cell carcinomas; *n* = 18) died from the disease and all but one were alive at the end of the follow-up. Exclusion of the Hürthle cell carcinomas did not alter the outcome of the univariate analyses (Fig. 1b).

## Discussion

Many protocols advocate treatment of MI-FTC by total thyroidectomy and ablative radioiodine, as this is the most effective strategy for detecting recurrent and/or metastatic disease [17, 18]. This strategy allows detection of otherwise invisible metastases by whole-body scintigraphy and thyroglobulin measurements, as well as early detection of recurrent disease. FTCs of Hürthle cell type are also usually treated by total thyroidectomy, based on observations of worse prognosis and sometimes less iodine uptake in many series [19]. Still, the superiority of this strategy has not yet been demonstrated in randomised studies; in the present study we rely on retrospective cohort data and consensus statements [17–22]. The optimal treatment of MI-FTC is more often a matter for discussion, as many series show excellent outcome also after lobectomy only [3]. In this series of MI-FTC, no specific treatment protocol was applied. However, significantly more patients with vascular invasion were treated by total thyroidectomy. Subsequently, patients with tumours showing vascular invasion more often received radioactive iodine ablation. Hence, clinical decision-making was often based on the presence of vascular invasion. Still, this more extensive treatment was not found to improve the outcome. Today, the National Swedish Guidelines, which are based on the ETA Guidelines, call for surgical treatment by total thyroidectomy of all FTCs [17, 23]. Based on our observations and the available literature, we propose that the rationale for treating MI-FTC with isolated capsular invasion by total thyroidectomy could be questioned.

In this retrospective study, we confirm some results from other studies of MI-FTC (Table 2). Many of the poor prognostic factors co-vary, making it difficult to identify single factors. In our study, no patient under the age of 50 at the time of diagnosis died from the disease. Also, all patients with tumours smaller than 3 cm survived. All five patients who died from the disease had tumours with both capsular and vascular invasion. Isolated capsular invasion fulfils the criteria for MI-FTC but still seems to correlate to an indolent prognosis. However, in a recent study, based on 251 patients with MI-FTC followed for median 7.2 years, distant metastases were observed in 12 % of patients with capsular invasion only [22]. It should, however, be stressed that the diagnostic evaluation of follicular thyroid neoplasias requires a stringent macroscopic evaluation of the tumour, and gross handling of the specimen demands the acquisition of multiple sections of the tumour capsule. As all patients in our series who succumbed to FTC displayed evidence of both limited vascular and capsular invasion, one cannot entirely rule out the possibility that these tumours did in fact display widely invasive behaviour, although not adequately represented in the blocks obtained. The extent of surgery and radioiodine ablation did not affect the prognosis. Concordant with our findings, age at diagnosis and the existence of metastatic disease were identified as important prognostic factors.

**Table 2:** Minimally invasive follicular thyroid cancer (MI-FTC) outcome and prognostic factors implicated in this and in relevant studies

First author/year/ref	*N*	Geographical region	Follow-up (years)	Outcome DM/Met	Summary of results
This study	58	Sweden	11.7	5/5	Risk of death from MI-FTC related to combined capsular and vascular invasion, age at surgery ≥50 years and male gender
Thompson et al. /2001/[3]	95	US	16.5	4/1	The prognosis of MI-FTC is excellent and lobectomy is sufficient for initial surgical management
O’Neill et al. /2011/[20]	98	Australia	3.3	7/1	Risk is related to age; vascular invasion is a risk factor; hemithyroidectomy is adequate for patients <45 years
Sugino et al./2012/[22]	251	Japan	7.2	54/14	Risk of death from MI-FTC is related to age ≥45 years and tumour size ≥4 cm
Goffredo et al. /2013/[25]	1200	US	16.8	6/2	Neither extent of surgery nor radioiodine treatment had any impact on survival; thyroid lobectomy is adequate

*N* number of MI-FTC cases, *DM* distant metastases, *DFS* death from disease

In this study, it is not possible to judge the effects of adjuvant treatment as those receiving radioiodine were a selected group of patients. Four of the five patients who died from MI-FTC had received ablative therapy. Among these, one patient treated with thyroidectomy was not radically operated, judging from the histopathological report. One may postulate that radioactive ablation does not replace the need for radical surgery. In a previous population-based nested case–control study of 1159 patients with differentiated thyroid cancer, half of whom died from their disease, we showed that radical removal of the primary tumour was an important prognostic factor [24]. However, the type or extent of surgery and postoperative radioiodine did not influence survival [24, 26]. Recent data from the SEER’s database indicate that patients with MI-FTC have survival comparable to that of the normative U.S. general population [25]. However, this database does not provide detailed information on histopathological and clinical characteristics. The study’s conclusion that MI-FTC can be treated as a benign lesion is challenged by the present and other studies [20, 22]. We instead propose that treatment should be modified based on the presence of vascular invasion, at least in elderly patients.

Surprisingly, all patients with Hürthle cell MI-FTC did well, although they are too few to support any distinct conclusions. Still, exclusion of these patients from the analysis did not alter the outcome (data not shown).

In conclusion, this study indicates that MI-FTC should not be regarded as an entirely indolent tumour. This is especially true for tumours with combined vascular and capsular invasion in patients over 50 years of age at diagnosis. The extent of the surgical procedure seems to be of less prognostic importance. Modification of the surgical strategy should rely on prognostic features.

## References

[1] Davies L, Welch HG (2014). Current thyroid cancer trends in the United States. JAMA Otolaryngol. Head Neck Surg..

[2] Chow SM, Law SC, Mendenhall WM, Au SK, Yau S, Yuen KT, Law CC, Lau WH (2002). Follicular thyroid carcinoma: prognostic factors and the role of radioiodine. Cancer.

[3] Thompson LD, Wieneke JA, Paal E, Frommelt RA, Adair CF, Heffess CS (2001). A clinicopathologic study of minimally invasive follicular carcinoma of the thyroid gland with a review of the English literature. Cancer.

[4] Lundgren CI, Hall P, Ekbom A, Frisell J, Zedenius J, Dickman PW (2003). Incidence and survival of Swedish patients with differentiated thyroid cancer. Int. J. Cancer.

[5] Delellis RA, Lloyd RV, Heitz PU, Eng C (2004). Tumours of the thyroid and parathyroid. WHO Classification of Tumours: Pathology and Genetics of Tumours of Endocrine Organs.

[6] Lo CY, Chan WF, Lam KY, Wan KY (2005). Follicular thyroid carcinoma: the role of histology and staging systems in predicting survival. Ann. Surg..

[7] Kroll TG, Sarraf P, Pecciarini L, Chen CJ, Mueller E, Spiegelman BM, Fletcher JA (2000). PAX8-PPARgamma1 fusion oncogene in human thyroid carcinoma (corrected). Science.

[8] Dwight T, Thoppe SR, Foukakis T, Lui WO, Wallin G, Höög A, Frisk T, Larsson C, Zedenius J (2003). Involvement of the PAX8/peroxisome proliferator-activated receptor gamma rearrangement in follicular thyroid tumors. J. Clin. Endocrinol. Metab..

[9] Learoyd DL, Messina M, Zedenius J, Robinson BG (2000). Molecular genetics of thyroid tumors and surgical decision-making. World J. Surg..

[10] Foukakis T, Au AY, Wallin G, Geli J, Forsberg L, Clifton-Bligh R, Robinson BG, Lui WO, Zedenius J, Larsson C (2006). The Ras effector NORE1A is suppressed in follicular thyroid carcinomas with a PAX8-PPARgamma fusion. J. Clin. Endocrinol. Metab..

[11] Wang N, Liu T, Sofiadis A, Juhlin CC, Zedenius J, Höög A, Larsson C, Xu D (2014). TERT promoter mutation as an early genetic event activating telomerase in follicular thyroid adenoma (FTA) and atypical FTA. Cancer.

[12] Trovato M, Fraggetta F, Villari D, Batolo D, Mackey K, Trimarchi F, Benvenga S (1999). Loss of heterozygosity of the long arm of chromosome 7 in follicular and anaplastic thyroid cancer, but not in papillary thyroid cancer. J. Clin. Endocrinol. Metab..

[13] Ghossein RA, Hiltzik DH, Carlson DL, Patel S, Shaha A, Shah JP, Tuttle RM, Singh B (2006). Prognostic factors of recurrence in encapsulated Hurthle cell carcinoma of the thyroid gland: a clinicopathologic study of 50 cases. Cancer.

[14] Nikiforov YE, Ohori NP, Hodak SP, Carty SE, LeBeau SO, Ferris RL, Yip L, Seethala RR, Tublin ME, Stang MT, Coyne C, Johnson JT, Stewart AF, Nikiforova MN (2011). Impact of mutational testing on the diagnosis and management of patients with cytologically indeterminate thyroid nodules: a prospective analysis of 1056 FNA samples. J. Clin. Endocrinol. Metab..

[15] Haugen BR, Alexander EK, Bible KC, Doherty GM, Mandel SJ, Nikiforov YE, Pacini F, Randolph GW, Sawka AM, Schlumberger M, Schuff KG, Sherman SI, Sosa JA, Steward DL, Tuttle RM, Wartofsky L (2016). 2015 American thyroid association management guidelines for adult patients with thyroid nodules and differentiated thyroid cancer. Thyroid.

[16] Cox DR (1972). Regression models and life tables. J. R. Stat. Soc. B.

[17] Pacini F, Schlumberger M, Dralle H, Elisei R, Smit JW, Wiersinga W (2006). European consensus for the management of patients with differentiated thyroid carcinoma of the follicular epithelium: European thyroid cancer taskforce. Eur. J. Endocrinol..

[18] Sosa JA, Udelsman R (2006). Total thyroidectomy for differentiated thyroid cancer. J. Surg. Oncol..

[19] Kuo EJ, Roman SA, Sosa JA (2013). Patients with follicular and Hurthle cell microcarcinomas have compromised survival: a population level study of 22,738 patients. Surgery.

[20] O’Neill CJ, Vaughan L, Learoyd DL, Sidhu SB, Delbridge LW, Sywak MS (2011). Management of follicular thyroid carcinoma should be individualised based on degree of capsular and vascular invasion. Eur. J. Surg. Oncol..

[21] Goffredo P, Jillard C, Thomas S, Scheri RP, Sosa JA, Roman S (2015). Minimally invasive follicular carcinoma: predictors of vascular invasion and impact on patterns of care. Endocrine.

[22] Sugino K, Kameyama K, Ito K, Nagahama M, Kitagawa W, Shibuya H, Ohkuwa K, Yano Y, Uruno T, Akaishi J, Suzuki A, Masaki C, Ito K (2012). Outcomes and prognostic factors of 251 patients with minimally invasive follicular thyroid carcinoma. Thyroid.

[23] Dionigi G, Kraimps JL, Schmid KW, Hermann M, Sheu-Grabellus SY, De Wailly P, Beaulieu A, Tanda ML, Sessa F (2014). Minimally invasive follicular thyroid cancer (MIFTC): a consensus report of the European Society of Endocrine Surgeons (ESES). Langenbecks Arch Surg..

[24] Lundgren CI, Hall P, Dickman PW, Zedenius J (2007). Influence of surgical and postoperative treatment on survival in differentiated thyroid cancer. Br. J. Surg..

[25] Goffredo P, Cheung K, Roman SA, Sosa JA (2013). Can minimally invasive follicular thyroid cancer be approached as a benign lesion?: a population-level analysis of survival among 1,200 patients. Ann. Surg. Oncol..

[26] Lundgren CI, Hall P, Dickman PW, Zedenius J (2006). Clinically significant prognostic factors for differentiated thyroid carcinoma. Cancer.

